# 

**Published:** 1895-07

**Authors:** 


### CONTENTS.

COMMUNICATIONS.

A History of the Mississippi Valley Dental Society........................ 313

COMMENCEMENTS.

Baltimore College of Dental Surgery ........................................ 354
Ohio College of Dental Surgery.............................................. 355
Pennsylvania College of Dental Surgery...................................... 355
Philadelphia Dental College.................................................. 356
New York College of Dentistry............................................... 358
Missouri Dental College...................................................... 358
Indiana Dental College....................................................... 359
Chicago College of Dental Surgery .......................................... 359
Cincinnati College of Dental Surgery........................................ 360

EDITORIAL.

American Dental Association.................................................. 361
To the Dentists of the United States........................................ 361
The Tri-State Dental Meeting................................................. 363

NOTICES.

Northern Iowa Dental Society................................................. 364
American Dental Association..................................................

